# The Black Esophagus in the Renal Transplant Patient

**DOI:** 10.1155/2019/5085670

**Published:** 2019-07-25

**Authors:** Michael Andrew Yu, Ramzi Mulki, Julia Massaad

**Affiliations:** ^1^Emory University, Department of Internal Medicine, USA; ^2^Emory University, Division of Digestive Diseases, USA

## Abstract

Acute esophageal necrosis (AEN) is an uncommon disease characterized by gastrointestinal bleeding and endoscopic findings of circumferential black-colored necrosis of the distal esophagus. Patients at risk include elderly males over the age of 65, who typically have multiple chronic medical issues including vascular disease and diabetes. Mortality is reported to be 32%. Here, we present a case of AEN in a renal transplant patient and describe potential inciting factors such as immunosuppression and opportunistic diseases.

## 1. Introduction

Black esophagus, commonly known as acute esophageal necrosis (AEN), is a rare clinical condition typically resulting from a combination of ischemic insult, caustic injury from gastric contents, and impaired mucosal protection [1–3]. It is most commonly observed in older males with multiple comorbidities, and has a reported overall mortality of 32% [1]. Patients classically present with hematemesis or melena, with esophagogastroduodenoscopy (EGD) findings of circumferential, black-colored diffuse necrosis at the distal esophagus terminating sharply at the gastroesophageal junction [1, 2]. Treatment includes maintaining hemodynamic stability, antimicrobial therapy, and proton pump inhibition [1, 2].

Interestingly, several reports of AEN have been described in renal transplant patients [4–9]. This particular group is especially vulnerable to gastrointestinal complications from opportunistic infections and mucosal injury due to immunosuppression [10, 11]. CMV esophagitis has been shown to be source of AEN and responds well to antiviral treatment [4, 5]. Transplant patients are often on chronic corticosteroids which increases the risk of peptic ulcer disease, or mycophenolate mofetil (MMF) which has been linked to increased GI bleeding by slowing intestinal cell turnover; this effect is heightened with concomitant NSAID use [10, 12, 13]. AEN has also been diagnosed in the immediate posttransplant period in the setting of hypoperfusion states from hemodynamic compromise and delayed graft function [6, 7]. In the literature, graft versus host disease and graft rejection have also been described as a culprit for AEN [8, 9]. Here, we describe a renal transplant patient on immunosuppression who developed AEN following vascular bypass surgery for limb ischemia.

## 2. Case Report

A 35-year-old male with history of end stage renal disease from hypertensive nephrosclerosis status post kidney transplant complicated by a right external iliac artery dissection, recurrent acute cellular rejection, and recurrent parvovirus infection presented with worsening chronic right foot pain. He was found to have right lower extremity limb ischemia due to right external iliac artery pseudoaneurysm with distal thromboembolism. He was started on heparin and on hospital day 3, underwent successful revascularization of his R lower extremity. No blood products were needed. Upon completion, heparin was continued. Notably, of his transplant immunosuppression regimen of MMF, tacrolimus, and prednisone, he had only been receiving tacrolimus from admission to his procedure, and was subsequently started on IV methylprednisolone and MMF. Post-procedural white blood cell count and hemoglobin were 20,000/mL and 12.5 g/dL, respectively.

**Figure 1:**
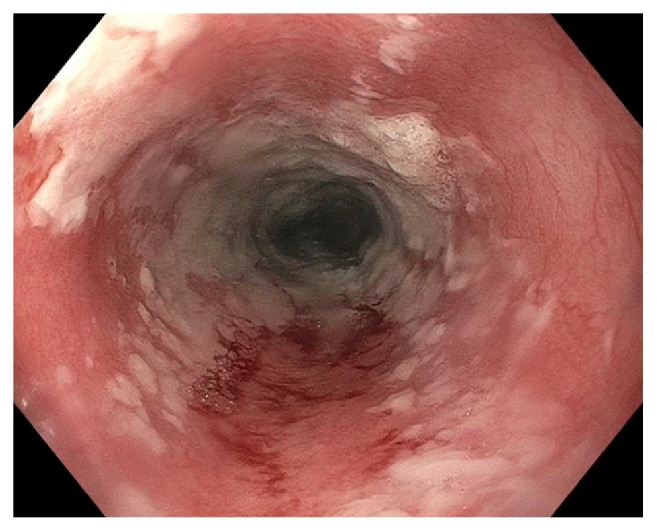
LA Grade D esophagitis extending to the middle esophagus.

**Figure 2:**
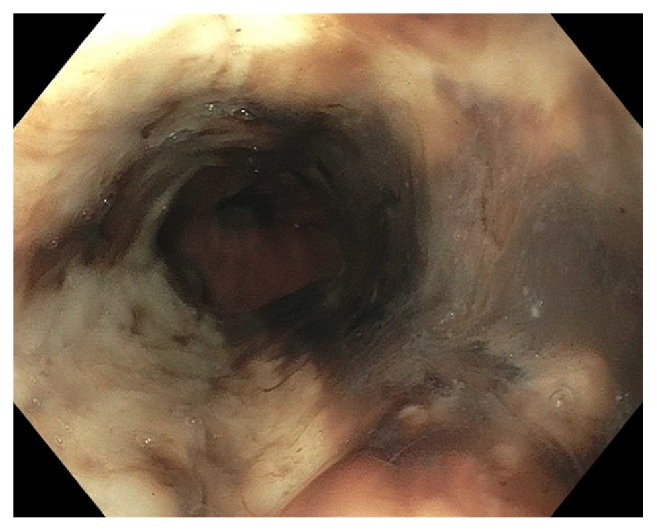
Necrotic appearing mucosa of distal esophagus.

**Figure 3:**
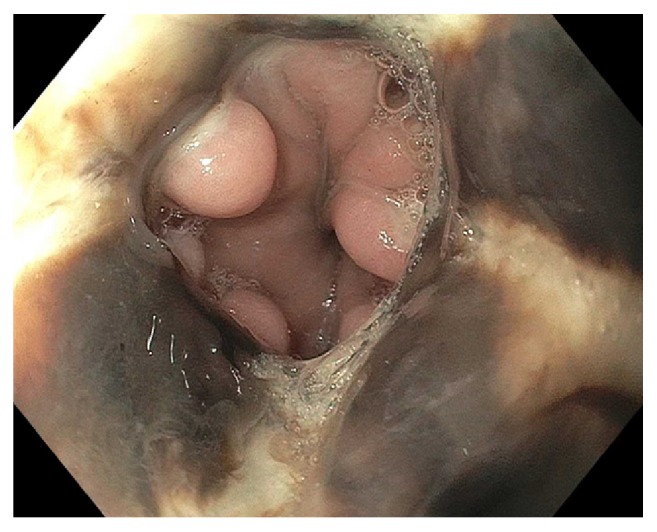
Necrotic appearing mucosa of distal esophagus.

**Figure 4:**
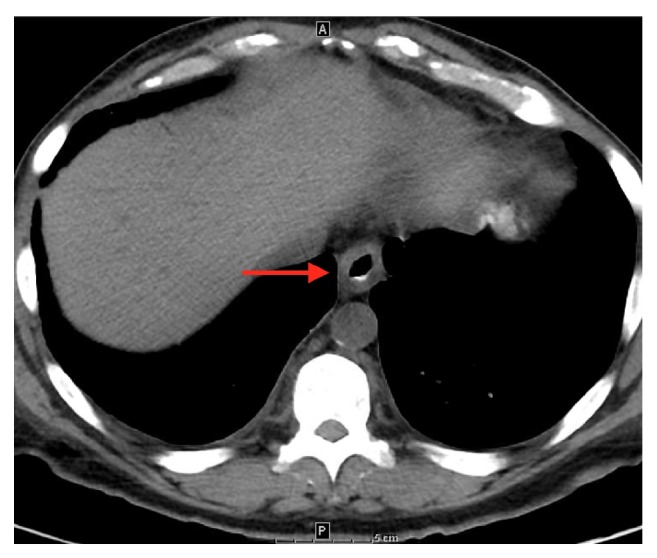
CT scan showing circumferential thickening of the distal esophagus.

Starting postop day (POD) 2, patient developed persistent nausea and hiccups and was made nil-per-os. He was tachycardic but afebrile and normotensive, and was started on broad spectrum antibiotics. By POD3, patient complained of abdominal pain and was without bowel movements since prior to surgery. Leukocytosis worsened to 25,400/mL and hemoglobin decreased to 9.6 g/dL. A CT abdomen/pelvis showed expected postsurgical changes without acute abnormalities. Patient received an aggressive bowel regimen and on POD4, had 3 episodes of melena. A hemoglobin checked afterwards was 7.3 g/dL. Heparin was discontinued and 1 unit of packed red blood cells was transfused with subsequent hemoglobin of 9.0 g/dL. Intravenous pantoprazole was started. On POD5, he underwent EGD, which showed LA Grade D esophagitis in the mid and distal esophagus. (Figure 1) There was necrotic appearing mucosa in the distal esophagus. (Figures 2 and 3) The mid esophagus was friable and bled on contact with the gastroscope. Endoscopic biopsy was deferred. Anticoagulation was resumed and patient was treated with sucralfate for 8 weeks and pantoprazole twice per day for 8 weeks. CMV level on PCR was undetectable. A later CT chest/abdomen showed circumferential thickening of the distal esophagus. (Figure 4). Melena resolved and patient tolerated a clear liquid diet. He completed a 7-day course of antibiotics and was bridged to coumadin. He was discharged following an unremarkable remaining hospital course. Notably, patient reported taking aspirin 81 daily and a prior ibuprofen use. On one-month follow up, patient was well with white blood cell count of 5,900/mL and hemoglobin of 10.9 g/dL.

## 3. Discussion

Black esophagus or AEN is a rare disease often seen in frail elderly males with multiple comorbidities including alcohol use disorder, peripheral vascular disease, diabetes, atherosclerosis, and cardiovascular and renal disease [1, 2]. It is thought to be multifactorial, likely caused from a mix of hemodynamic compromise leading to tissue hypoperfusion, corrosive injury from stomach contents, and impaired mucosal defense [1, 2]. Classically, the diagnosis is made based on signs and symptoms of GI bleeding, leukocytosis, and EGD findings of diffuse, circumferential necrosis at the distal esophagus [1, 2]. Accepted treatment include making the patient nil-per-os, maintaining optimal organ perfusion, treating any infection with antibiotics, and starting adequate acid suppressing agents such as a PPI or antihistamines [1, 2].

This renal transplant patient has multiple issues making him especially vulnerable to AEN. First, patients with kidney disease have a higher chance of developing peripheral vascular disease, another risk factor for AEN [14]. In patients undergoing renal transplants, there is increased risk of ischemic injury from circulatory instability, and AEN has been described as a complication in the immediate post-transplant period [6, 7]. Next, these patients are prescribed various immunosuppressing agents like corticosteroids and MMF which compromise the gastrointestinal mucosa ability to maintain effective barrier function and give risk to opportunistic infections such as CMV esophagitis [4, 5, 10]. Finally, graft rejection has also been described to cause AEN, likely through hemodynamic compromise [8, 9].

Our case highlights the various risk factors a renal transplantation patient possesses for developing AEN. The patient was admitted for limb ischemia due to complication of the right external iliac artery dissection from renal transplant but was likely aggravated by peripheral vascular disease of renal disease. He underwent revascularization of his right lower limb which added a component of hemodynamic compromise. Gastrointestinal mucosal barrier compromise from chronic steroids, MMF, and NSAIDs probably also exacerbated his risk of AEN. It is unlikely that the patient's history of acute cellular rejection played a role.

Extra vigilance is needed when caring for renal transplant patients. Certain immunosuppression agents have gastrointestinal complications and should be carefully selected and coupled with acid suppressing agents. If AEN is considered, esophagitis from opportunistic infections must be ruled out. EGD during the post-transplant period or after major procedures may also be considered, especially if there was significant hemodynamic insult.
